# The Preparation of Monomer Casting Polyamide 6/Thermotropic Liquid Crystalline Polymer Composite Materials with Satisfactory Miscibility

**DOI:** 10.3390/polym14204355

**Published:** 2022-10-16

**Authors:** Mingmin Li, Jiahao Qiu, Yifei Yue, Jingbing Liu, Baohua Zhang

**Affiliations:** 1 National-Certified Enterprise Technology Center, Kingfa Science and Technology Co., Ltd., Guangzhou 510705, China; 2 Guangzhou Key Laboratory of Sensing Materials & Devices, Center for Advanced Analytical Science, c/o School of Chemistry and Chemical Engineering, Guangzhou University, Guangzhou 510006, China; 3 Faculty of Materials and Manufacturing, Beijing University of Technology, Beijing 100124, China

**Keywords:** block copolymers, polyamide 6, compatibility, liquid-crystalline polymer (LCP), morphology

## Abstract

It is highly expected to develop a simple and effective method to reinforce polyamide 6 (PA6) to enlarge its application potential. This is challenging because of frequently encountered multi-component phase separations. In this paper, we propose a novel method to solve this issue, essentially comprising two steps. Firstly, a kind of poly (amide-block-aramid) block copolymers, i.e., thermotropic liquid crystalline polymer (TLCP)-polyamide 6 (TLCP-PA6), that contains both rigid aromatic liquid crystal blocks, and flexible alkyl blocks were synthesized. It is unique in that TLCP is chemically linked with PA6, which is advantageous in excellent chemical and physical miscibility with the precursors of monomer casting polyamide 6 (MCPA6), i.e., ε-caprolactam. Secondly, such newly synthesized block copolymer TLCP-PA6 was dissolved in the melting ε-caprolactam, and followed by in situ polymerization to obtain composite polymer blends, i.e., MCPA6/TLCP-PA6. The thermodynamic, morphological, and crystalline properties of MCPA6/TLCP-PA6 can be easily manipulated by tailoring the loading ratios between TLCP-PA6 and ε-caprolactam. Especially, at the optimized condition, such MCPA6/TLCP-PA6 blends show an excellent miscibility. Systematic characterizations, including nuclear magnetic resonance (NMR), Fourier-transform infrared spectroscopy (FT-IR), differential scanning calorimeter (DSC), and polarizing optical microscope (POM), were performed to confirm these statements. In view of these results, it is anticipated that the overall mechanical properties of such PA6-based polymer composites will be satisfactory, which should enable applications in the modern plastic industry and other emerging areas, such as wearable fabrics.

## 1. Introduction

Polyamide 6 (PA6) is well known as high performance engineering plastic with high strength, good fatigue resistance, and satisfactory wearing durability [1,2,3,4]. However, its poor notched impact toughness (especially at low temperatures) restricts its overall performance, which limits its applications in modern plastic industries and newly emerging fields [5,6,7,8,9], e.g., wearable fabrics [4], self-supporting water purification films [9], and nanofiltration membrane [7], etc. To solve this issue, PA6 matrix has been modified by reinforcing materials, such as hydrogenated acrylonitrile-butadiene-styrene (ABS) [10], styrene-co-acrylonitrile (SAN) [11], hydrogenated styrene-butadiene-styrene (SEBS) [12], polyamide 66 [13], poly(tetrafluoroethylene) (PTFE) [14], and thermotropic liquid crystalline polymer (TLCP) [15], etc. For those reinforcing monomers or polymers, they have similar functional groups to that of PA6, which is advantageous to obtain compatible polymer composites to overcome the phase separation problem at the interface of PA6 and reinforcing additives through chemical and physical interactions or adhesions between them.

As we know, among those polymer modifiers [10,11,12,13,14,15], TLCP is one promising choice. Because of the peculiar rigid-rod chemical structures, TLCP in the solid form possesses many unique properties, e. g., low melt viscosity and high mechanical modulus in the oriented direction. It is convenient to obtain polymeric composites with much enhanced mechanical properties by blending amorphous or crystalline polymers with TLCP. Accordingly, many studies have focused on PA6 composites reinforced with TLCP [16,17,18,19,20]. However, when TLCP was introduced into PA6 matrix, the resulting performance of those composite was unsatisfactory in general due to poor compatibility between those species. To solve this dilemma, previous solutions were mainly divided into two categories, i.e., introducing the compatibilizer into the TLCP/PA6 blending system or modifying the chemical structure of TLCPs. For example, Tjong et al. [21,22] reported a well compatible TLCP/PA6 blend by adding maleic anhydride grafted polypropylene (MAP) or random styrene–maleic anhydride copolymer (RSMA). Shin and Chung disclosed that the adhesion between the TLCP and PA66 was enhanced by introducing a long flexible spacer in the mainchain of TLCP [23]. Castellano et al. suggested to introduce bisphenol-A type epoxy coupler into the reactive blend of Nylon 6,6 and Vectra A (one kind of TLCP), which increased the melt viscosity of Nylon matrix via chain extension and branching process and thus obtain compatible Nylon/TLCP block copolymers via such coupling agents [24]. Moreover, back in 1990, Pawlikowski et al. reported a successful synthesis of A-B-A type segmented copolymers (A: rigid TLCP blockes; B: flexbile PA blocks) by solution polycondensation method, which was based on special reactive monomers and an optimized loading ratios of them [25]. Despite this progress, it is noticed that effective strategies toward achieving compatible PA6/TLCPs composites with satisfactory properties are scarce. To enlarge the applications of such engineering plastics, it is urgent to explore many more approaches.

**Figure 1:**
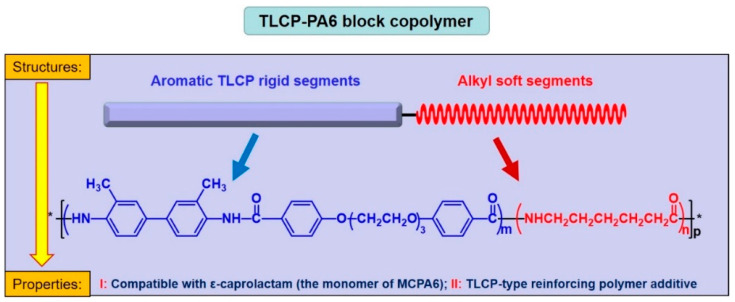
Chemical structure and properties of TLCP-PA6 block copolymer in this work.

In this paper, we present a novel method to prepare well compatible PA6/TLCP composite, which shows good flexibility and scalability. In detail, the matrix-type polymer (PA6) is introduced into the main chain of TLCP as the spacer to improve the compatibility between the TLCP and the precursor of MCPA6, i.e., ε-caprolactam, which is followed by in situ copolymerization to achieve MCPA6/TLCP-PA6 composite. Firstly, a new thermotropic liquid crystalline polyamide block copolymer (TLCP-PA6) (see Figure 1) is designed and synthesized, which contains rigid blocks of liquid crystal segments and flexible blocks of PA6. After that, a series of MCPA6/TLCP-PA6 composites with different blending ratios are prepared by in situ anionic polymerization of ε-caprolactam with dissolved TLCP-PA6. Systematic characterizations were performed, which confirmed the excellent compatibility of these MCPA6/TLCP-PA6 composites and its application potentials.

## 2. Materials and Methods

### 2.1. Materials

The sodium hydroxide (NaOH) and toluene-2,4-diisocyanate (TDI) (analytical reagent) were purchased from Beijing Chemical Reagents Company (Beijing, China) and used without further purification. Bis(4-carboxyphenyl) ether (OT) (analytical reagent) was obtained from Beijing Yinhai Chemical Reagents Company (Beijing, China). ε-Caprolactam was obtained from Beijing Qingshengda Chemical Reagents Company (Beijing, China). 4,4ʹ-diamino-3,3ʹ-dimethylbiphenyl (PEG_3_) was prepared according to the earlier report [26]. OT and PEG_3_ were dried under vacuum at 80 °C. N-methyl-2-pyrrolidone (NMP) (Acros, Shanghai, China) was dried in vacuum over CaH_2_ and stored with molecular sieves. Pyridine was bought from Beijing Chemical Reagents Company (Beijing, China) and used as received. All other reagents were obtained from commercial sources and used without further purification.

### 2.2. Synthesis of Thermotropic Polyamide Liquid Crystalline Block Copolymers (TLCP-PA6)

**Scheme 1:**
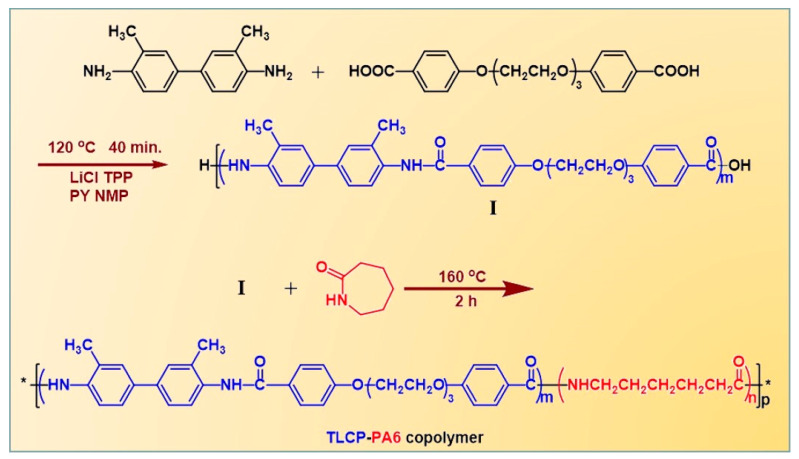
Schematic illustration of the synthesis of TLCP-PA6 block copolymer.

The synthesis route of the TLCP-PA6 block copolymer is schematically shown in Scheme 1. In detail, it was performed as follows: a mixture of PEG_3_ (2.3 mmol), diamines (2.3 mmol), triphenyl phosphite (TPP, 1.5 g), and LiCl (0.25 g) in pyridine (2.5 mL) and NMP (10 mL) was heated at 120 °C (bath temperature) for 40 min with stirring. ε-caprolactam (0.69 mmol) was then added into the mixture and heated up to 160 °C (bath temperature) for another 2 h with stirring. After cooling, the reactive mixture was diluted with N, N-dimethylformamide (DMF) and poured into methanol to extract the polymer. It was washed in boiling methanol and dried under vacuum for 48 h (gray, yield = 84%). As a control, thermotropic homopolyamide liquid crystalline polymer (TLCP) was synthesized by copolycondensation of PEG_3_ and OT, which referred to the reported method by Higashi et al. [27]

### 2.3. Preparation of MCPA6/TLCP-PA6 Blends

Under nitrogen atmosphere, TLCP-PA6 was dissolved in melted ε-caprolactam monomer at 170 °C to achieve a homogeneous transparent solution in which different loading ratios were used to obtain a series of MCPA6/TLCP-PA6 blends with different blending ratios. The mixture was exposed to vacuum at 170 °C for approximately 20 min to remove trace water. After that, NaOH (0.2 wt. %) was added under stirring and then the mixture was exposed to vacuum for another 10 min. Subsequently, TDI (0.4 wt. %) was added and the final mixture was polymerized at 170 °C for 1 h. After polymerization, the products were cooled to room temperature and extracted with boiling water to eliminate the residual monomer. The MCPA6/TLCP-PA6 blends were dried in a vacuum oven for 24 h at 80 °C. Finally, the MCPA6/TLCP-PA6 blends with the different content of TLCP-PA6 (e.g., 0~30%) were obtained.

### 2.4. Measurement

The intrinsic viscosity ([η]) of TLCP-PA6 was measured in NMP/LiCl (5 wt. %) at a concentration of 0.1 g∙dL^−1^ with a suspended level Ubbelohde viscometer in which it was thermostated at 30 ± 0.1 °C, using the one-point intrinsic viscosity method.

^1^H nuclear magnetic resonance (^1^H-NMR) spectra of TLCP-PA6 were measured by an equipment of Bruker 400 MHz, i.e., preparing sample solutions in D_2_SO_4_ and using 3-(trimethylsilyl) propanesulfonic acid sodium salt (DSS) as an internal standard.

Fourier-transform infrared spectroscopy (FTIR) spectra ranging from 4000 to 500 cm^−1^ were measured for these polymers by using a Nicolet 5700 spectrometer and potassium bromide (KBr) (Xi’an Qiyue Biotechnology Co., LTD, Xi’an, China) as the window plate.

The measurements of polarizing optical microscope (POM) were carried out with a polarizing microscope (MELTING POINT BY-2, Beijing, China) equipped with a heating stage (YAZAWA) (Tokyo, Japan).

Scanning electron microscope (SEM) (FEI Quanta 2000, Hillsboro, OR, USA) was used to characterize the cryo-fractured surfaces of all these MCPA6/TLCP-PA6 blends. Specimens were prepared as follows. Firstly, the specimens were fractured in liquid nitrogen. Secondly, the surfaces of the samples were etched with NMP for 24 h at room temperature to remove TLCP-PA6 domains and then dry thoroughly, which was followed by sputtering gold onto the etched surfaces prior to SEM examinations.

Differential scanning calorimeter (DSC) were carried out on a SCINCO S-650 (Shanghai, China) equipment. All measurements were protected under nitrogen atmosphere, starting at room temperature and using a heating rate of 10 °C∙min^−1^. The samples were first heated to 260 °C and maintained for 3 min. to erase thermal history, which were followed by a cooling step at the rate of 5 °C∙min^−1^. The crystallization and melting thermograms were taken from the subsequent cooling and the second heating cycles. The crystallinities of the polyamide parts of those samples were determined according to the following equation:

Xc_(DSC)_ = ΔH_f_/fΔH_f_* × 100% (1)

where ΔH_f_ is the melting enthalpy of MCPA6 in the sample, f is the homopolymer weight fraction in the blend and ΔH_f_* is the melting enthalpy of the matrix polymer of 100% crystallization (ΔH_f_ = 230 J∙g^−1^). T_c,0_ is the initial temperature of crystallization in the cooling scan and used for evaluating the nucleating effect [28].

Wide angle X-ray diffraction (WAXRD) was performed on an X-ray diffraction analyzer Bruker AXS (D8 ADVANCE, Karlsruhe, Germany) equipped with a rotating Cu anode generator system using Cu Kα-alpha1 (λ = 1.5406 Å) radiation. The diffraction angles (2θ) were measured ranging from 5° to 40°. The data were accumulated for 6 s at angular intervals of 2θ = 0.1°.

## 3. Results

### 3.1. Structure and Liquid Crystalline Analysis of TLCP-PA6

**Figure 2:**
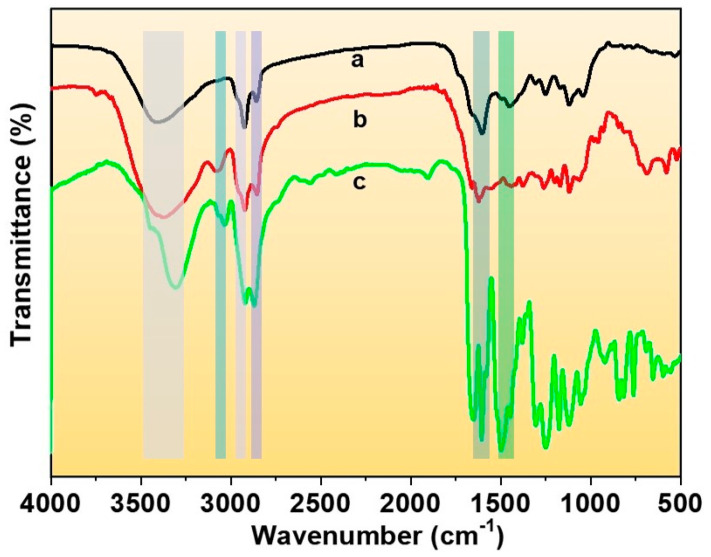
FTIR spectra of different samples, i.e., (**a**) pure MCPA6, (**b**) MCPA6/TLCP-PA6 blend (100 wt. %/15 wt. %) and (**c**) pure TLCP-PA6, respectively.

The chemical structure of TLCP-PA6 is confirmed by ^1^H NMR (Figure S1) and FTIR spectroscopy (Figure 2). As it is shown in Figure S1, the spectral assignments of ^1^H NMR clearly support the proposed structure of TLCP-PA6 sample. The area from the absorption of the protons in the TLCP-PA6 is in the range of 1.0~9.0 ppm. In the FTIR spectrum of TLCP-PA6 (curve c in Figure 2), the specific FTIR bands are attributed to the stretching vibration of hydrogen-bonded N-H (3316 cm^−1^), N-H connected to amide II (3035 cm^−1^), amide I, II (1652 cm^−1^, 1499 cm^−1^), and methylene (2922 cm^−1^), respectively.

**Figure 3:**
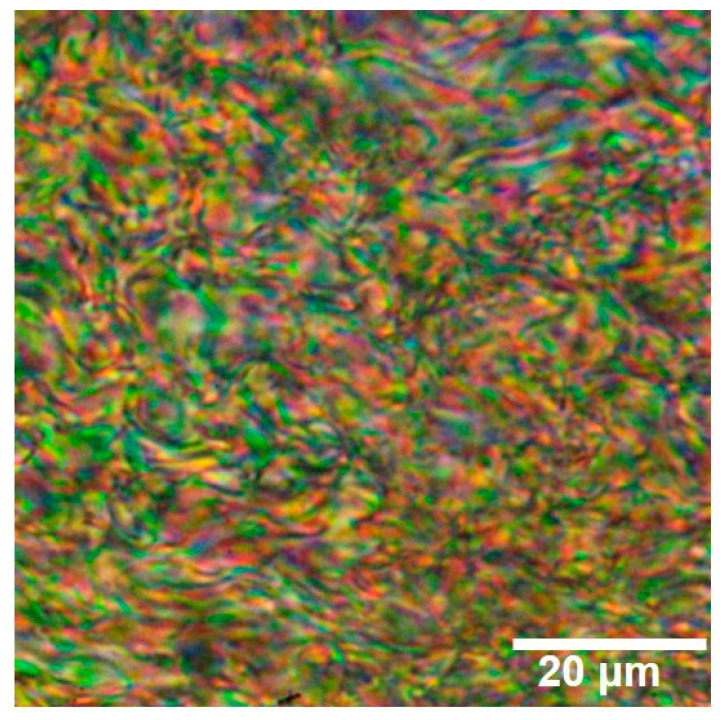
The POM image of TLCP-PA6 at 300 °C (cooling from 340 °C, cross polarizers).

The mesomorphic properties of TLCP-PA6 were confirmed by the measurements of POM using a heating-cooling stage. Phase transition temperatures and the corresponding enthalpy values were obtained by DSC (Figure S2) and summarized in Table 1. As observed, TLCP-PA6 shows the characteristics of liquid crystalline on heating or cooling. In the heating cycle, it clearly melts into mesophase at ca. 290 °C at which the temperature was distinctly lower than the melting point temperature of TLCP (T_m_ = 311.4 °C). After that, it turns to isotropic at ca. 350 °C. Such a temperature is also lower than the clearing temperature of TLCP (T_i_ = 371.7 °C) as seen optically. These results are consistent with those observed by DSC (Table 1). Moreover, It is found that the [η] of TLCP-PA6 is far higher than that of TLCP. This phenomenon should be ascribed to the copolymerization of TLCP and PA6. As for the measurements of POM in the cooling scan, TLCP-PA6 displays isotropic liquid crystal behaviors. As shown in Figure 3, this TLCP-PA6 block copolymer shows nematic phase in a wide temperature range, e.g., 290~350 °C.

### 3.2. Morphology

As for TLCP-PA6 and MCPA6, we notice that only TLCP-PA6 dissolves well in NMP. Therefore, NMP was used to etch the MCPA6/TLCP-PA6 blends, with the purpose of rendering the observed morphology as unambiguous as possible. As there are no voids for the NMP-treated sample, it is assumed that chemical interactions should take place between TLCP-PA6 and MCPA6 component [29,30].

**Figure 4:**
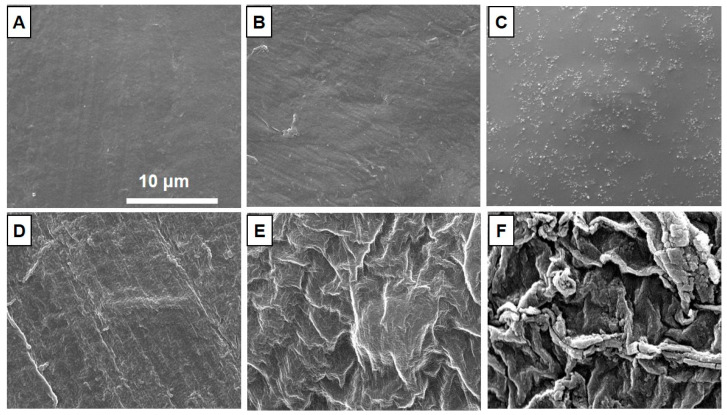
SEM images of fractured surfaces of MCPA6 (100 wt. %)/TLCP-PA6 (x wt. %) containing different contents of TLCP-PA6, i.e., x wt. % = 0% (**A**), 2.5 wt. % (**B**), 10 wt. % (**C**), 15 wt. % (**D**), 20 wt. % (**E**), and 30 wt. % (**F**).

Figure 4 presents the SEM images of the fractured surfaces of those MCPA6/TLCP-PA6 blends. Apparently, when the content of TLCP-PA6 within the MCPA6/TLCP-PA6 blends is in the range of 2.5~10%, the dispersed phase is dispersed as nearly spherical nanoparticles within the MCPA6 matrix (Figure 4B,C). For the MCPA6/TLCP-PA6 blend sample containing 10% TCLP-PA6, the diameter of dispersed nanoparticles is approximately 100 nm. The SEM images (Figure 4B,C) also show the good dispersion of TLCP-PA6 within the MCPA6 matrix. As it is shown (Figure 4B,C), the phase boundary between them is very blurred. As the content of TLCP-PA6 is further increased to 15~20%, those morphological features of nanoparticle-dispersion disappear. By contrast, those MCPA6/TLCP-PA6 blends turn to a homogeneously surface morphology (Figure 4D,E). It is speculated that hydrogen bonding and/or amide reactions between TLCP-PA6 and PA6 are greatly enhanced as increasing the content of TLCP-PA6. These strong interactions should further enhance the compatibility of those two phases and render the phase boundary more blurred. Additionally, their fractured surfaces are rougher as compared with those MCPA6/TLCP-PA6 blends merely containing 2.5 and 10% TLCP-PA6. When the content of TLCP-PA6 is further increased to 30%, the MCPA6/TLCP-PA6 blends exhibit a layered structure (Figure 4F). To understand it, we notice that in this condition the anionic polymerization is found to be too fast and the melt viscosity of the mixture is too high. It is speculated that gas generated in the reaction process cannot be discharged in time, which may result in such distinguished morphological features.

Therefore, the analysis concerning SEM evolutions indirectly indicates that at a wide blending range (e.g., less than 30%) the compatibility of MCPA6/TLCP-PA6 blend at the phase interface is satisfactory. There is no obvious phase separation between these two components. It is believed that at least two reasons should account for it. Firstly, since the chemical structures of MCPA6 and TLCP-PA6 are similar, they intrinsically possess good physical compatibility (similarity-intermiscibility principle). Secondly, TLCP-PA6 interacts with MCPA6 during the in situ polymerization of ε-caprolactam monomers. Such chemical interactions further help to overcome the phase separation issue.

### 3.3. FTIR Analysis

Figure 2 shows the FTIR spectra of MCPA6, TLCP-PA6, and MCPA6/TLCP-PA6 (100 wt. %/15 wt. %) blends, respectively. As shown in Figure 2 and Table 2, FTIR stretching vibrations of hydrogen-bonded NH, NH connected to amide II, and amide I, II of the MCPA6/TLCP-PA6 blend are quite different from those of the MCPA6 counterpart. As for the MCPA6/TLCP-PA6 (100 wt. %/15 wt. %) blend, the stretching vibration peak of hydrogen-bonded NH shifts over 30 cm^−1^ to the lower wavenumbers, which is between that of MCPA6 and TLCP-PA6. In addition, the stretching vibration peak of NH connected to amide II is present in the FTIR of the MCPA6/TLCP-PA6 (100 wt. %/15 wt. %) blend. It should be ascribed to the formation of the new hydrogen bonding and/or an amide reaction between TLCP-PA6 and MCPA6 [31]. The CH_2_ asymmetrical and symmetrical stretching vibrations of the MCPA6/TLCP-PA6 (100 wt. %/15 wt. %) blend are almost the same as those of the MCPA6 counterpart. However, the intensity ratio of them is different. From the above analysis, it is also suggested that the chemical interaction should occur between MCPA6 and TLCP-PA6 in the MCPA6/TLCP-PA6 blended composites.

### 3.4. Thermal Properties

**Figure 5:**
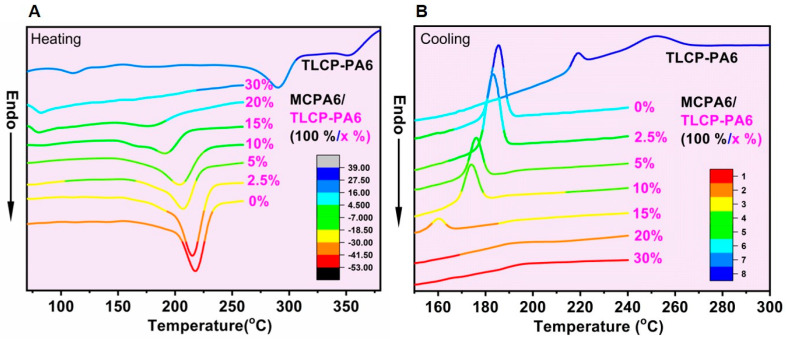
Thermograms of different MCPA6/TLCP-PA6 samples (100 wt. %/x wt. %), x wt. % = 0~30 wt. %, i.e., (**A**) heating curves and (**B**) cooling curves.

Figure 5 shows the cooling and heating DSC scans of pure TLCP-PA6 and MCPA6/TLCP-PA6 blends (100 wt. %/x wt. %) with different content of TLCP-PA6 (x wt. %). The melting and crystallization parameters of these samples are summarized in Table 3. By increasing the amount of TLCP-PA6 in MCPA6/TLCP-PA6 blends, it is found that T_m_, ΔH_f_, T_c,m_, T_c,0_, ΔH_c_, and Xc_(DSC)_ of MCPA6 in MCPA6/TLCP-PA6 blends are monotonically decreased. It is interesting to note that in the case where 30 wt. % TLCP-PA6 is added, there is no endothermic peak. While 20 wt. % or 30 wt. % TLCP-PA6 is added, the exothermic peak disappears, but both glass transition peaks appear. The T_g_ of those MCPA6/TLCP-PA6 blends is increased by increasing the content of TLCP-PA6. However, neither of them shows the lower T_g_ value, compared to pure TLCP-PA6. Combined, all of those results indicate that TLCP-PA6 greatly influences the thermal dynamic properties of those MCPA6/TLCP-PA6 blends because of its fine dispersion and its chemical interaction with MCPA6 during polymerization progress. The strong physical and chemical interaction improves the compatibility between MCPA6 and TLCP-PA6. Additionally, the finer the dispersion, the more energy is consumed in spherulite growth, i.e., the decreased T_c,0_ in phenomena [32]. On the other hand, it is believed that a strong interaction between MCPA6 and TLCP-PA6 and the increased melt viscosity of the resultant MCPA6/TLCP-PA6 blend are combined to retard the crystallization of MCPA6. Therefore, T_c,m_ of MCPA6/TLCP-PA6 blends is decreased by increasing the content of TLCP-PA6. Compared to pure MCPA6, the crystalline peaks of MCPA6/TLCP-PA6 blends are broadened, which indicates that the crystallinity of MCPA6 in MCPA6/TLCP-PA6 blends is decreased and the crystallization is becoming incomplete.

### 3.5. XRD Analysis

**Figure 6:**
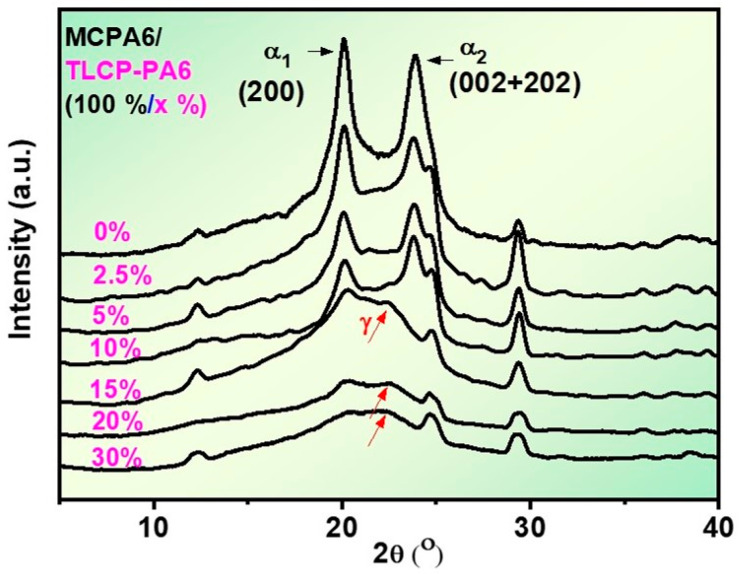
WAXRD results of MCPA6/TLCP-PA6 blended samples (100 wt. %/x wt. %) with the different content of TLCP-PA6 (x wt. % = 0~30 wt. %).

Figure 6 shows the XRD patterns of these MCPA6/TLCP-PA6 blends (100 wt. %/x wt. %) containing different content of TLCP-PA6 (x wt. % = 0~30 wt. %), which reflects the changes in the crystalline structure of those blends. When the content of TLCP-PA6 is in the range of 0~10 wt. %, all of these MCPA6/TLCP-PA6 blends display α-form crystals in which two main crystalline peaks, i.e., α_1_ and α_2_, correspond to the crystallographic planes of (200) and (002 + 202), respectively [33,34]. With increasing the content of TLCP-PA6, these two crystalline peaks gradually decrease in intensity. In addition, such a decreasing tendency is more evident for the α_2_ form. When the content of TLCP-PA6 is increased to 15 wt. %, the α_1_ peak becomes indistinct and the α_2_ peak disappears. Meanwhile, the γ-form crystal is clearly present at this condition [35]. It is well known that the impact strength of an MCPA6 sample containing γ-form crystals is remarkably higher than that of the MCPA6 counterpart with α-form crystals. It is because chain interactions in the γ-form are weaker than those in the α-form [36]. When the content of TLCP-PA6 is 20 wt. % and 30 wt. %, a broad amorphous halo is observed. It indicates that the amorphous region in those composite materials is dominant. Among all of these MCPA6/TLCP-PA6 blends, there is a shoulder on the right side of the α_2_ peak. Combined, all of these suggest that the incorporation of TLCP-PA6 copolymer significantly influences the crystalline structure of the MCPA6 in those MCPA6/TLCP-PA6 blends. The degree of crystallinity of these blends is decreased by increasing the content of TLCP-PA6. Meanwhile, the diffraction pattern of all those blends shows an interesting feature, i.e., a small crystalline peak at 2θ = 29.4°, which is analogous to earlier reports [32,37]. In view of the complexity of crystalline diffraction peaks of this polymer blend, we still cannot thoroughly designate such a crystal structure and reflection plane. A more in-depth study will be focused on this point and disclosed elsewhere.

## 4. Conclusions

In this study, a new kind of thermotropic liquid crystalline polyamide block copolymer (TLCP-PA6) is synthesized and fully characterized. It shows liquid crystalline (nematic phase) in a wide temperature range (290~350 °C) and is capable of fine dissolving in ε-caprolactam. Accordingly, in situ anionic polymerization of ε-caprolactam with TLCP-PA6 is successfully performed and then a series of MCPA6/TLCP-PA6 blends with different blending ratios are obtained. The results of SEM images indicate that at a low loading content of TLCP-PA6 (2.5~10 wt. %), it is well dispersed as nanoparticles in the MCPA6/TLCP-PA6 blends. While in the range of 15~20 wt. %, such nanoparticle features disappear and it turns to a homogeneous morphology without an evident two-phase boundary. While the content of TLCP-PA6 is 30 wt. %, the blends display the layered structure. In short, MCPA6/TLCP-PA6 blends with a suitable loading content of TLCP-PA6 (e.g., less than 30 wt. %) are well compatible composites, which is due to strong physical and chemical interactions between these two components (confirmed by FTIR and thermomechanical analysis). The results of DSC measurements show that various parameters including ΔH_f_, T_m_, ΔH_c_, T_c,m_, T_c,0_, and Xc_(DSC)_ of MCPA6 in MCPA6/TLCP-PA6 blends are significantly decreased by increasing the content of TLCP-PA6. In addition, introducing the moiety of TLCP-PA6 into the MCPA6 matrix is confirmed to influence the crystalline of MCPA6, as verified by WAXRD measurement. Especially, while the content of TLCP-PA6 is higher than 15 wt. %, a satisfactory γ-form crystalline of PA6 appears for these blends, which can enhance the impact strength of the composites. By contrast, MCPA6/TLCP-PA6 blends with a lower content of TLCP-PA6 merely display the well-known α_1_- and α_2_-form crystals. In short, these novel explored MCPA6/TLCP-PA6 composites are expected to be an advanced reinforcing engineering plastic material with an excellent mechanical performance. Further mechanical evaluations and practical applications are under consideration and will be reported in future work.

## Figures and Tables

**Table 1:** Intrinsic viscosities and thermal properties of TLCP and TLCP-PA6.

Samples	[η] (dl^.^g^−1^)	DSC (First Heating)			
		T_g_ (°C)	T_m_ (°C)	T_i_ (°C)	H_m_ (J∙g^−1^)
TLCP	1.30	120.0	311.4	371.7	54.8
TLCP-PA6	4.04	110.8	291.9	352.3	91.1

**Table 2:** FT-IR results of different samples.

Vibration Mode	TLCP-PA6	MCPA6/TLCP-PA6 (100 wt. %/15 wt. %)	MCPA6
Hydrogen-bonded NH stretching	3316	3378	3413
NH stretching connected to amide II	3035	3080	-
CH_2_ asymmetrical stretching	2922	2923	2923
CH_2_ symmetrical stretching	-	2855	2858
Amide I	1652	1623	1603
Amide II	1499	1452	1444

**Table 3:** Melting and crystallization parameters of different samples derived from the DSC measurements.

MCPA6/ TLCP-PA6 (wt. %/wt. %)	Heating (2nd)				Cooling		
	T_g_ (°C)	T_m_ (°C)	ΔH_f_ (J∙g^−1^ PA6)	Xc_(DSC)_ (%)	ΔH_C_ (J∙g^−1^ PA6)	T_c,m_ (°C)	T_c,0_ (°C)
100/0	-	217.3	−104.6	48.5	116.9	185.3	192.3
100/2.5	-	215.4	−93.6	41.7	112.6	183.0	190.3
100/5	-	206.9	−74.7	34.1	98.4	175.9	182.4
100/10	-	204.0	−70.9	33.9	88.5	174.0	181.4
100/15	-	190.9	−50.0	20.5	45.3	160.1	165.8
100/20	80.2	-	-	-	-	-	-
100/30	82.0	-	-	-	-	-	-

## Data Availability

The data presented in this study are available on request from the corresponding author.

## Supplementary Materials

The following are available online at https://www.mdpi.com/article/10.3390/polym14204355/s1, Figure S1: ^1^H NMR spectra of the TLCP-PA6 copolymer. Figure S2: DSC curves of TCLP and TLCP-PA6 samples (the first heating).
