# Supplementary material for: The Preparation of Monomer Casting Polyamide 6/Thermotropic Liquid Crystalline Polymer Composite Materials with Satisfactory Miscibility
Source: Polymers (Basel). 2022 Oct 16;14(20):4355. doi: 10.3390/polym14204355 (PMC9611747; doi:10.3390/polym14204355)
Supplement: Supplementary file 1 [file polymers-14-04355-s001.zip › polymers-1924743-supplementary.pdf]

*Supplementary Materials*

# The Preparation of Monomer Casting Polyamide 6/Thermotropic Liquid Crystalline Polymer Composite Materials with Satisfactory Miscibility

**Mingmin Li [1], Jiahao Qiu [2], Yifei Yue [2], Jingbing Liu [3,*] and Baohua Zhang [2,*]**

1 National-certified Enterprise Technology Center, Kingfa Science and Technology Co., Ltd., Guangzhou 510705, China
2 Guangzhou Key Laboratory of Sensing Materials & Devices, Center for Advanced Analytical Science, c/o School of Chemistry and Chemical Engineering, Guangzhou University, Guangzhou 510006, China
3 Faculty of Materials and Manufacturing, Beijing University of Technology, Beijing 100124, China
* Correspondence: liujingbing@bjut.edu.cn (J.L.); ccbhzhang@gzhu.edu.cn (B.Z.)

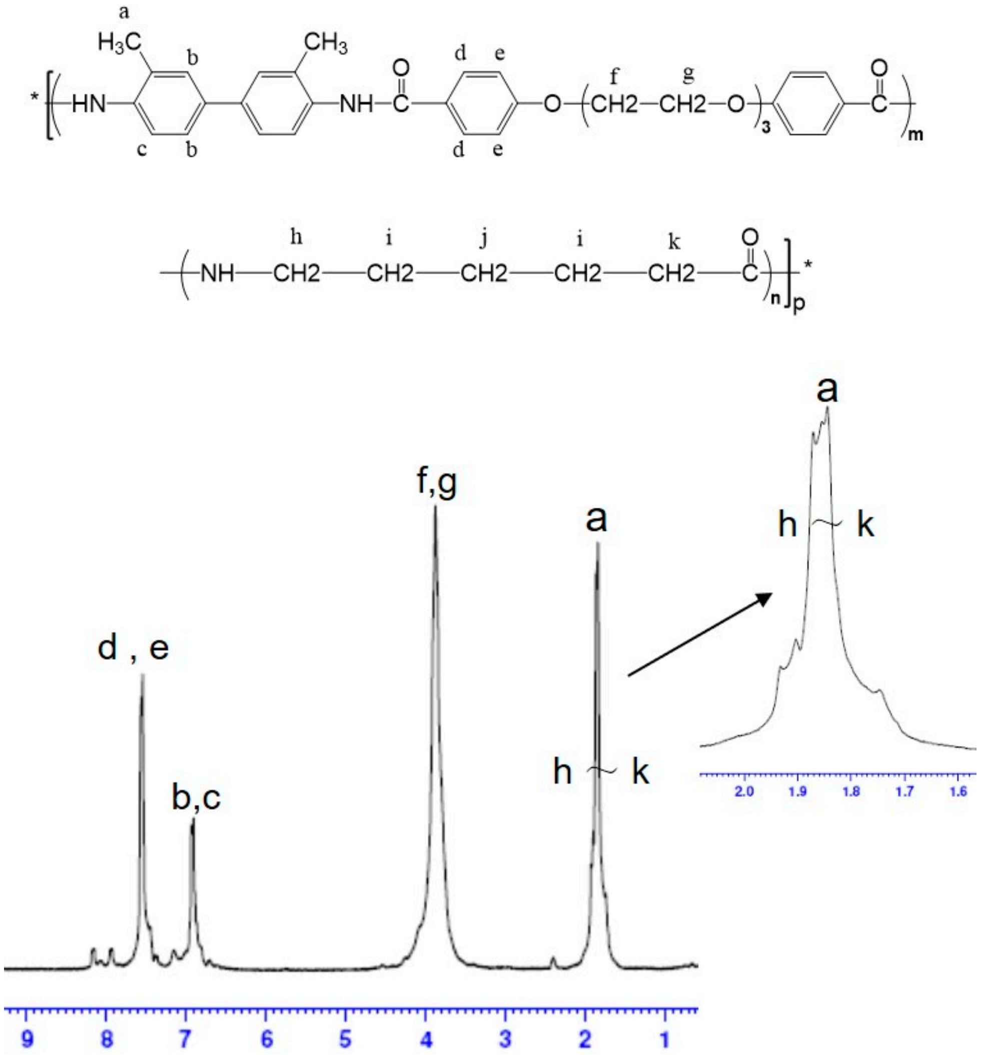


**Figure S1.** $^1$H NMR spectra of the TLCP-PA6 copolymer.

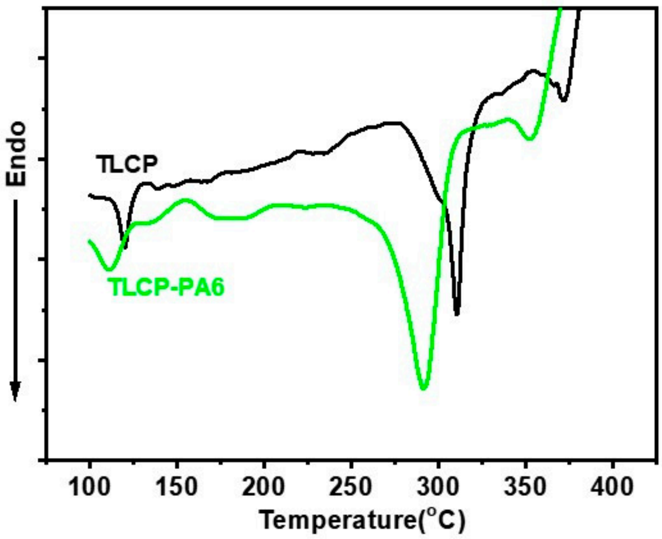


**Figure S2.** DSC curves of TCLP and TLCP-PA6 samples (the first heating).
